# Pigmented paravenous retinochoroidal atrophy (Review)

**DOI:** 10.3892/etm.2014.1648

**Published:** 2014-03-28

**Authors:** HOU-BIN HUANG, YI-XIN ZHANG

**Affiliations:** 1 Department of Ophthalmology, The General Hospital of Chinese PLA, Beijing 100853, P.R. China; 2 Department of Ophthalmology, Hainan Branch of General Hospital of PLA, Sanya, Hainan 572013, P.R. China

**Keywords:** retinitis pigmentosa, retinal pigment epithelium, human, ophthalmology, clinical, disease process

## Abstract

Pigmented paravenous retinochoroidal atrophy (PPRCA) is an uncommon disease characterized by perivenous aggregations of pigment clumps associated with peripapillary and radial zones of retinochoroidal atrophy that are distributed along the retinal veins. Patients are usually asymptomatic and the disease process is non-progressive or slow and subtly progressive. It is commonly bilateral and symmetric. The cause of the condition may be unknown or idiopathic, although a dysgenetic, degenerative, hereditary etiology or even an inflammatory cause has been hypothesized. A non-inflammatory cause is referred to as primary, while inflammation-associated PPRCA is referred to as secondary or pseudo PPRCA. The present study reviewed and summarized the features of PPRCA.

## 1. History and general background

Brown first described pigmented paravenous retinochoroidal atrophy (PPRCA) in 1937 in a 47-year-old man who developed alopecia areata (1). Since then there have been a few additional case reports (2–5) and to date >100 cases have been reported worldwide.

### Terminology and general introduction

PPRCA has appeared in literature under the appellations of retinochoroiditis radiata (1), pseudoretinitis pigmentosa following measles, chorioretinitis striata (6,7), congenital pigmentation of the retina (8), melanosis of the retina, paravenous retinal degeneration, pigmented paravenous chorioretinal degeneration (2), pigmented paravenous chorioretinal atrophy (9) and PPRCA (10). The generally accepted name is pigmented paravenous chorioretinal atrophy or PPRCA, which is preferred for the following reasons. The condition is characterized by retinal pigment epithelium (RPE), choriocapillaris atrophy and pigmentation along the retinal veins. A number of studies generally agree that the main region affected is the RPE (11–14). Thus, PPRCA involves the RPE primarily, with secondary atrophy of the underlying choroidal vasculature (10,15–17). Therefore, choriopathy is secondary to retinopathy and PPRCA is the more appropriate name. In addition, the descriptive term is relatively succinct and correctly reflects the current level of understanding of the disease process.

PPRCA is commonly bilateral and symmetric, but appears to have variable expressivity and a spectrum of mild to severe ophthalmoscopic changes (18,19). Certain reported cases have been markedly asymmetric or unilateral (20–23). Patients are usually asymptomatic and exhibit non-progressive or slow and subtle progression of the disease. Diagnosis is often fortuitously made during a routine examination and is based on a typical and characteristic fundus appearance. Family and medical histories are often non-contributory and the disease is more commonly found in young individuals. Gender predilection is not distinct (3).

### Etiology

The cause of the condition is unknown. In the majority of patients with PPRCA, the disease occurs sporadically, with the exception of specific cases of familial occurrence (16,19,20,24–28). Thus, a hereditary nature for this condition appears to be reasonable assumption. As a result, there has been varied speculation as to the inheritance by autosomal dominant, recessive, X-linked and Y-linked modes of transmission. However, there is no convincing evidence for any one of these transmission modes. Certain authors have proposed that the condition has a degenerative origin (10,15,29). Since specific patients have bilateral PPRCA with macular coloboma, ophthalmologists have postulated that it is a developmental abnormality in nature (30) and thus is a passing disorder of the retinal blood vessels during the embryonic stages of eye development (14). This view that PPRCA has a congenital origin has also received additional support (31). Furthermore, an inflammatory etiology has been hypothesized. The earliest case report indicated an inflammatory cause since the affected individual had tubercular spondylitis (1), while another early case report described a child with congenital syphilis (32). Following these early studies, a number of inflammatory PPRCA cases were reported with a variety of inflammatory causes, including Behçet’s disease (33), measles (34), rubeola (35), uveitis (11,36) and other unknown causes of inflammation (4,37). However, these cases that developed due to various inflammatory agents or other definite reasons, particularly inflammatory retinal vein vasculopathy resulting from various causes, are not real PPRCA but should be considered as a pseudo PPRCA.

## 2. Clinical features

### Presentation

The major complaints of patients with PPRCA are asymptomatic or mild blurred vision (38). Visual loss is usually mild and nyctalopia is not common. Certain patients who complained of poor dark adaptation, poor night vision or a blind eye did not suffer from PPRCA but instead had retinitis pigmentosa (RP) or pseudo PPRCA, as aforementioned (4,19,20,26,30,37). Particular observations in PPRCA have also been reported, including a shadow in front of the eye, diminished peripheral vision, mild pain and photopsia, involuntary closure of the upper eyelid, headaches and observing halos around lights (4,15,39). However, whether these observations are associated with the disease entity remains unknown.

### Visual acuity, visual field and color vision

The finding of PPRCA is usually incidental and does not affect vision. In specific cases, central visual acuity may be mildly reduced. Severely reduced vision has also been observed as a result of rare changes, including anisometropia amblyopia (19), cataracts (11), glaucoma (26,40), nystagmus (19,20), vitreous opacity (37) and macular involvement (detail *vide infra*). However, these changes may be observations specific to individuals rather than associations within the phenotype. Visual field changes may be normal. Depending on the topography of pigmentation and atrophy, the visual field may be manifested as a ring scotoma (20), a geographic scotoma, a quadrant defect (6), paracentral scotomas (4,17,20,26), concentric constriction (3,17,22,26,30,31,39), an enlarged blind spot (11,35) or scattered scotomas (41) corresponding to the atrophic paravenous areas. Color vision is usually normal, although mild non-specific dyschromatopsia or an acquired color vision defect has been observed in specific cases (13,26,30,31).

### Anterior segment and vitreous

In the majority of cases, anterior segment and vitreous examination reveals no significant features (4). As for the pseudo PPRCA cases secondary to inflammation or other causes, slitlamp examination of the anterior segment may reveal keratic precipitates (11), cells in the anterior chamber, iridic synechiae, cataracts (11), floaters in the anterior vitreous, marked cells and snowball opacities in vitreous (37) or dense vitreous opacity (11).

### Retinal observations

#### Retinal pigmentation and retinochoroidal atrophy

The typical funduscopic appearance is a bilaterally symmetrical accumulation of pigment and retinochoroidal atrophy distributed along the retinal veins, invariably beginning at a distance from the optic nerve head. Peripapillary pigmentary changes may be observed, as well as areas of chorioretinal atrophy adjacent to the perivenular pigmentary changes. The unaffected retinal areas appear to be normal. A number of fundus manifestations have been reported and asymmetrical or unilateral manifestations have been observed (23,25). The pigmentation is typical of bone corpuscle pigmentation, coarse pigment clumps and fine pigmentary changes. Retinochoroidal atrophy has been observed as subretinal yellowish lesions that manifest as small rounded flecks or contiguous circumscribed patches around the retinal vessels, coexisting with absent or depigmented pigment epithelium and pigment clumping that arborize and spread into the peripheral retina with the vasculature (19).

In the mild form of the disorder, there are only a few scattered areas with minimal evidence of retinochoroidal atrophy and paravenous pigmentation. In intermediate cases, bone spicule pigment accumulates around the majority of the veins and a minimal amount of retinochoroidal atrophy or regional retinochoroidal atrophy is observed. By contrast, marked cases are associated with peripapillary and posterior pole pigmentary sheen and diffuse areas of retinochoroidal atrophy adjacent to extensive and heavy paravenous pigment accumulation (18). It has been reported that with the progression of retinochoriopathy, the grayish lesion and RPE atrophy gradually enlarge and become associated with more pigmentation, possibly accompanied by further constriction of the peripheral visual fields (39). In addition, the choroidal vessels in the atrophic lesion become more clearly visible, presumably as a result of the progressive RPE atrophy (17). Areas of retinochoroidal atrophy, previously confined to a strict paravenous distribution, exhibit more extensive and frequent confluence with ‘islands’ of normal retina and specific areas demonstrate a distinctly scalloped appearance, resembling a posteriorly situated gyrate atrophy of the choroids. Finally, several localized atrophic areas with crystal deposition may be present in the peripheral retina (39). However, these reported cases may not be primary PPRCAs, but actually secondary cases.

#### Macula, optic disc and retinal vessels

In a minority of cases, the macula is affected. Macular changes include cystoid macular edema (37), star-shaped exudate (35), macular wrinkling (31), epiretinal membrane (6), macular pigmentary stippling (35), pigmentary macular degeneration (15,26), macular depigmentation (17), lamellar macular holes (42), macular dysplasia (43), macular RPE atrophy (17), central areolar macular atrophy (19), excavated macula (19) and macular coloboma (14,30,44). However, whether these macular observations are associated with the phenotype of the disease entity or distinct findings remains unknown.

The optic disc is normal in the majority of cases with no waxy pallor of the optic disc or frank optic nerve atrophy. Gliotic disc or peripapillary gliosis has been reported (19) and optic disc drusen may be present with typical PPRCA (13). Since disc drusen usually has autosomal dominant inheritance and is known to be associated with RP, Young and Small (13) postulated that concomitant PPRCA and disc drusen may represent a syndrome expressed by contiguous genes or may be caused by the coinheritance of associated traits, unassociated traits, sheer coincidence or retinal degeneration.

In the majority of cases, the caliber of the retinal vessels is normal, but attenuated retinal arteries and arteriolar attenuation with sheathing have been occasionally reported (6,19,30,35).

#### Other observations

Other observations of the fundus have been reported in addition to those aforementioned, including abnormal retinal sheen in the area of the posterior pole (19), localized atrophic areas with crystal deposition in the peripheral retina (39) and peripheral tangential vitreous traction (31). PPRCA is rarely associated with vitreoretinal degeneration even in children, however, liquefaction of the vitreous and vitreous cells, vitreal condensation (20), posterior vitreous detachment and collapse, dense opacity and retinal lattice degeneration (19) have been reported in PPRCA patients. A rare and significant observation of retinal microangiopathy has been reported (22). Microaneurysms and telangiectasia of the retinal vessels were observed in the temporal periphery and later developed into hard intraretinal exudates. Fluorescein angiography revealed areas of capillary nonperfusion, arteriovenous communications, microaneurysmal dilatation and vessel wall staining. Limaye and Mahmood postulated that retinal photoreceptor damage may induce retinal microangiopathy and that the photoreceptor damage may be secondary to the primary degeneration of the RPE (22). However, retinal microangiopathy may be not associated with the phenotype.

#### Angiography

Fundus fluorescein angiography (FFA) shows transmitted hyperfluorescence or chorioretinal atrophy, depending on the severity of the lesion. In one study (17), in a mild forms of the condition, FFA showed diffuse window defects with hyperfluorescence, consistent with RPE degeneration, and blockage of fluorescence in the areas of pigment clumping along the retinal vessels. In the more severe early arterial phase, FFA showed an extensive area of choriocapillaris atrophy with prominently visible choroidal vessels along the major retinal veins adjacent to the disc. In the arteriovenous phase, delimited hyperfluorescence was observed at the edge of the atrophic area, with hypofluorescence corresponding with the areas of pigment migration. No fluorescein leakage beneath or within the neuroretina was observed at any stage.

Indocyanine green angiography (ICGA) has disclosed hypofluorescence in all phases and demonstrated that hypofluorescence covers the atrophic lesions and partly extends into the areas that are hyperfluorescent with fluorescein. This indicates that choriocapillaris atrophy is underestimated by FFA and evaluation may be improved with ICGA (17).

#### Fundus autofluorescence (FAF) and optical coherence tomography (OCT)

Fleckenstein *et al* (45) observed arcs of increased FAF with a crescent-like distribution surrounding the area of RPE atrophy in a 29-year-old man with bilateral PPRCA. In the left eye, the central macula was surrounded by a ring of increased FAF that was broadened at the temporal side. Microperimetric assessment revealed normal light sensitivity in the central macula and severely reduced light sensitivity in areas demarcated by the arc of increased FAF. Simultaneous confocal scanning laser ophthalmoscopy and high-resolution spectral domain OCT (SDOCT) imaging revealed that the line of increased FAF corresponded to the junction between a zone with preserved retinal layers on the SDOCT scan and a zone where the presumed external limiting membrane (ELM) appeared to rest directly on the RPE layer. In a broadened area of the left eye with increased FAF, the interface of the inner/outer segments of the photoreceptors (IPRL) was not present. However, the distance between the RPE layer and the presumed ELM appeared to be increased compared with that in the areas with normal-appearing FAF and the loss of IPRL (45). In another case, SDOCT scans of the veins revealed thinning of the retinal layers with increased backscattering and disorganization of the RPE-choriocapillaris complex. Hyperreflective plaques with underlying shadowing corresponded to the pigment clumps observed clinically (42).

### Visual electrophysiology and dark adaptometry

Electrodiagnostic data are variable and nonspecific, ranging between normal and mildly affected, even markedly subnormal or a totally extinguished electroretinogram (ERG) and normal and abnormal electrooculogram (EOG) (11,37). This variation in observations may be due to the varying ages of the patients and the severity of disease. Alternatively, the variation may signify that several conditions can present in this manner (20).

ERGs disclose abnormal or low-normal rod responses (scotopic ERG), cone responses (photopic ERG), maximal combined responses, flicker responses and oscillatory potentials. B-wave amplitude reduction is the most common observation, followed by A-wave amplitude reduction and delayed latency (16,35,37,46). In specific cases, ERGs have revealed a decreased scotopic response with a normal photopic response and vice versa in other patients (19,35). These results indicate a selective loss of rod or cone function. However, scotopic ERG observations have not been found to correlate with night blindness. Certain patients did not experience nyctalopia, but had scotopic ERGs with reduced amplitude (20). It was hypothesized that scotopic responses reflect the size and extent of atrophic lesions, while the bright flash and photopic responses apparently did not parallel changes in lesion size. In one eye of a patient that did not experience ophthalmoscopic changes, the photopic ERG was abnormal. In other patients with marked asymmetrical fundus changes, symmetrical ERG responses were observed, despite one eye being morphologically normal and the contralateral eye showing moderate to severe changes (19). The variation in the reduction of bright flash and photopic responses may reflect the heterogeneous impairment of various cell types in the retina. In a patient with poor night vision, pattern ERGs showed a reduced N95 amplitude, while pattern visual evoked potential (PVEP) revealed a low to normal amplitude and latency. These pattern ERG and PVEP observations indicate ganglion cell involvement (37).

EOGs have also shown variable ratios of light peak/dark trough voltage (normal, borderline and abnormal). The reduction in light rise results from a dysfunction of RPE and photoreceptor cells. A marked reduction in the light peak/dark trough (L/D) ratio may result, at least in part, in impaired photoreceptor function, as shown by ERG. Studies have indicated that EOGs may be normal in severely affected patients and abnormal in cases with mild lesions (11,20). Such variability of EOGs may also reflect the heterogeneity of the disease involving a variety of cell types in the retina and RPE. Yonemura *et al* (47) demonstrated that hyperosmolarity responses were more frequently abnormal than the L/D ratio and were particularly useful for the early diagnosis of the disorder. However, improved evaluation of neurosensory retinal dysfunction may be conducted with multifocal ERG.

Dark adaptation curves are usually normal with monophasic and elevated final rod thresholds (6,15,30). However, certain patients with abnormal dark adaptation have no night vision problems.

### Complications and systemic association

Other ocular problems have been reported as complications to PPRCA, including angle closure glaucoma (16,26,40), nystagmus (19,20), hypermetropia (13,19,40), hyperopic astigmatism (31), anisometropia (13,19,24), amblyopia (19,24), convergent squint (26), esotropia (19,40) and exotropia (6). However, these problems may only be particular observations and not associations within the phenotype.

Subtle systemic features were observed in three members of a family in the form of bifid uvulae or mild loose-jointedness (19), and vitreoretinal degeneration was present in the children with PPRCA; there is a definite association between vitreoretinal degeneration and generalized connective tissue disorders, including submucous clefts, as observed in Stickler syndrome. Brown also reported alopecia areata (1).

## 3. Histopathology and attributes

The basic histopathological change, according to angiographic and electrophysiological results, is atrophy of the RPE and choriocapillaris, including depigmentation and pigmentation. Fluorescein studies in young patients with this entity revealed RPE atrophy with preservation of the choroidal vasculature underlying the atrophic RPE (10). In patients with a more advanced stage of the disease, choroidal atrophy has been observed in the involved areas (15). Consequently, PPRCA primarily involves RPE, with secondary atrophy of the underlying choroidal vasculature (16). Such relative sparing of the choriocapillaris in early stages of the disease is also a feature of RP (17,20). Therefore, PPRCA may be considered as an additional, incomplete, self-limited form of RP (24). PPRCA develops similarly to a number of diseases that are acquired or inherited and produce the funduscopic appearance of RP (31). Traversi *et al* reported a case supporting this hypothesis (24) in a family with unilateral RP and macular involvement in the mother and hereditary PPRCA in the daughter and son. The observations from this case clearly indicated that it was RPE damage that had been transmitted in an autosomal dominant manner with variable expression. Thus, PPRCA is a RPE disorder, in addition to RP, and has a heterogeneous hereditary nature. An additional family provided circumstantial evidence for this hypothesis (25).

Histopathological characteristics of PPRCA were verified by Linek *et al* (48) who described funduscopic observations in PPRCA in five dogs observed over a period of 24 years. The five dogs had localized hyperreflectivity in the tapetal fundus and a characteristic perivascular distribution along specific peripheral retinal blood vessels. In these areas, geographic copper-brown coloration was present that tended to become darker with time. Ophthalmoscopic signs of inflammation were lacking. Funduscopic abnormalities slowly progressed in size over the years. The most important histopathological observation was that one dog revealed severe retinal atrophy with multifocal perivascular distribution, mainly affecting the tapetal fundus and occasionally expanding into the nontapetal fundus. These results further indicate that the main region to be affected in PPRCA is the RPE.

Noble and Carr also hypothesized that PPRCA is an individual entity, distinct from RP. RP is an inherited generalized retinal dystrophy, usually symptomatic early in life and progressive in nature. Perivenular bone corpuscular pigment is only one of a number of fundus abnormalities that may occur. PPRCA is not a generalized disorder. It is slowly progressive, if at all, and has pigmentation only along the distribution of the veins (18).

## 4. Diagnosis and differential diagnosis

The diagnosis of PPRCA is based on typical and characteristic fundus appearance. FFA, ICGA, electrophysiological tests and visual fields may confirm the diagnosis. A study disclosed that fundus autofluorescence imaging is able to reveal the exact extent of neurosensory dysfunction, which may exceed the dimensions anticipated by conventional examinations (49). Laboratory studies include chest roentgenogram, complete and differential blood cell counts, serum electrolytes, serum protein electrophoresis, erythrocyte sedimentation rate, tuberculin skin test (50), antinuclear antibody, C-reactive protein, venereal disease research laboratory test, serum antibody tests for herpes simplex virus I and II, herpes zoster virus, cytomegalovirus, rubella and measles and serological tests associated with syphilis, toxoplasmosis, systemic lupus erythematosus or rheumatoid arthritis.

Differential diagnoses include chorioretinal degeneration and inflammatory diseases that cause chorioretinal atrophy, including RP (pericentral, sector and typical), helicoid peripapillary chorioretinal atrophy, serpiginous choroidopathy, angioid streaks, cone dystrophy or degeneration, Stickler syndrome, gyrate atrophy choroideremia, Wagner’s dominant vitreoretinal degeneration, sarcoidosis, syphilis, acute retinal necrosis, cytomegalovirus retinitis, tuberculous disseminated choroiditis, onchocerciasis, toxoplasmosis, frosted branch angiitis and various disorders that are termed pseudoretinitis pigmentosa.

### Pericentral RP

Pericentral RP, also known as pericentral pigmentary retinopathy (51–53), peripapillary (pericentral) pigmentary retinal degeneration (dystrophy) (12,54–56), peripapillary pigmentary degeneration, pericentral retinal dystrophy or annular (pericentral, circinate) pigmentary retinal dystrophy (57–59), is a rare, localized chorioretinal disorder. The disorder is characterized by bilateral, usually symmetrical, transparency of the RPE associated with bone spicule pigment clumping in a peripapillary distribution with a nasal extension to the disk and temporally in an arcuate fashion. Although the majority of changes follow the vessel arcades, there are a number of involved areas unassociated with the distribution of the retinal vasculature. In one study, ophthalmoscopic examination revealed a segmental, grayish metallic sheen in association with bone spicule pigmentation radiating from the disk along the temporal vessel arcades and joining temporal to the macula. The optic disk, retinal vessels, periphery and macula appeared to be normal. This was indicated to be a segmental disease of the RPE-photoreceptor complex by visual function tests and fluorescein angiography, which revealed the presence of visual field scotomas corresponding to the distribution of the retinal lesion, decreased retinal sensitivity in abnormal retinas and normal sensitivity in ophthalmoscopically unaffected areas, a slight reduction in the ERG with normal latencies, normal EOG light rise and pigment transmission defects with hyperfluorescence in the affected retinas, and normal fluorescence in the adjacent retina (58). The mean rates of remaining ocular function loss in patients with pericentral RP are generally slower compared with those in patients with typical forms of RP (58). The trait may be autosomal recessive or dominant. However, in certain cases, a positive family history cannot be found (58).

### Helicoid peripapillary chorioretinal degeneration

Also referred to as Sveinsson chorioretinal atrophy (60,61), helicoid peripapillary chorioretinal degeneration is a rare bilateral fundus disorder characterized by wing-shaped, well-defined, atrophic areas of the choriocapillaris and RPE, which radiate from the optic nerve head towards the macula and the fundus periphery. They are not associated with the retinal vessels, which are normal. No signs of an old or recent inflammatory process, including scarring and pigment clumping, are present but in certain cases slight pigmentation enhancement along the margins is observed. Only the white background of the sclera and large choroidal vessels is visible within the lesion. The floor is not ectatic and the overlying sensory retina exhibits no gliotic, dysplastic or edematous changes. Fluorescein angiography may reveal an early hypofluorescence due to the absence of the choriocapillaris and late staining of the degenerative lesions, but without leakage. This entity may be autosomal dominantly inherited or sporadic (62). A progressive tearing and retraction of the RPE or Burch’s membrane around the optic disk may be involved in the pathogenesis of the disorder. This tearing results primarily from dysplastic abnormalities of the RPE that surrounds the optic disk. Dystrophic lesions progress slowly and may affect the macula and even the peripheral retina (2,63–65).

### Serpiginous choroidopathy

Serpiginous choroidopathy, also known as geographic choroidopathy or geographic helicoid peripapillary choroidopathy, is a chronic, progressive and recurrent disease of the RPE, choriocapillaris and choroid (66,67). The disease characteristically involves the juxtapapillary retina and extends radially to involve the macula and peripheral retina. The active stage of serpiginous choroidopathy is known as serpiginous choroiditis (geographic choroiditis) (68,69), characterized by the presence of inflammatory signs, asymmetry of the fundus lesions, sharply demarcated gray-yellow lesions with irregular borders involving the RPE and choriocapillaris and pigment clumping. Fluorescein angiography may reveal leakage at the borders of the lesions. As the lesions resolve, the RPE, choriocapillaris and choroid atrophy. Fibrous tissue deposition and pigment epithelial hyperplasia and hypoplasia may occur. Vitritis, anterior uveitis and subretinal neovascularization have been shown to be associated with serpiginous choroiditis (70,71). The disease is usually bilateral and the cause is unknown.

### Angioid streaks

Angioid streaks are defined as a series of linear, cracked-line dehiscences of Bruch’s membrane, with secondary changes in the RPE and choriocapillaris. The streaks may be progressive or degenerative with varied presentation, color, distribution and retinal involvement. Angioid streaks may be accompanied by peripapillary and radial atrophy, with no direct association with the vessels (2,72,73).

## 5. Prognosis and treatment

PPRCA is usually non-progressive or slowly progressive (5). However, Yanagi *et al* (17) considered the disease to be stationary in younger patients and slowly progressive in older subjects. Although it has been argued that specific cases of PPRCA are progressive, these patients may have pseudo PPRCA (3,4,74,75). There is no specific treatment for RPE or choriocapillaris atrophy.

## 6. Summary and conclusions

PPRCA is a rare disorder that is not well understood or classified. PPRCA may be an individual entity or may be a subtype of RP, depending on the particular clinical characteristics. The disease is usually asymptomatic with only a fundus abnormality. In addition, PPRCA is non-progressive or slowly progressive. However, specific cases experience more symptoms and more severe visual loss, with more variable and severe fundus changes and other progressive ocular abnormalities. These patients may not have PPRCA, but actually have pseudo PPRCA, which is a similar comparison to that of pseudo RP with primary RP.

## Abbreviations

PPRCA: pigmented paravenous retinochoroidal atrophy
RPE: retinal pigment epithelium
RP: retinitis pigmentosa
FFA: fundus fluorescein angiography
ICGA: indocyanine green angiography
OCT: optical coherence tomography
SDOCT: spectral domain optical coherence tomography
ERG: electroretinogram
EOG: electrooculogram
PVEP: patterned visual evoked potential
ELM: external limiting membrane
IPRL: interface of inner and outer photoreceptor layers
FAF: fundus autofluorescence
